# Energy Poverty and Depression in Rural China: Evidence from the Quantile Regression Approach

**DOI:** 10.3390/ijerph19021006

**Published:** 2022-01-17

**Authors:** Jun Zhang, Yuang He, Jing Zhang

**Affiliations:** School of Agricultural Economics and Rural Development, Renmin University of China, No. 59 Zhongguancun Ave., Haidian Dist., Beijing 100872, China; heyuang@ruc.edu.cn (Y.H.); zhangjing2020@ruc.edu.cn (J.Z.)

**Keywords:** energy poverty, depression, rural Chinese, quantile regression

## Abstract

Despite the growing awareness and interest in the impact of energy poverty on depression, studies in developing economies are relative limited, and there is a gap of knowledge of such impact among rural individuals in China. In this study, we investigate the impact of energy poverty on depression among rural Chinese individuals aged 16 and above, and our sample includes 13,784 individuals from 6103 households. With data from the 2018 China Family Panel Studies, we apply the instrumental variable (IV) quantile regression approach to address the potential endogeneity of energy poverty and allow for heterogeneous effects of energy poverty on depression across individuals with different levels of depression. Our estimates from the IV quantile regression suggest a strong positive impact of energy poverty on depression at the upper quantile of depression scores, but no impact at the middle and lower quantiles. The primary results are robust and consistent with alternative energy poverty measures, and we find that energy poverty does not affect depression of low-risk individuals (with low depression scores), but it does affect that of high-risk individuals. We also find individual socio-demographic factors of age, gender, household size, religious belief, education, marriage and employment status play roles in affecting depression. The findings of this study generate policy implications for energy poverty alleviation and mental health promotion.

## 1. Introduction

Depression is a major public health concern around the world, and it is estimated that about 280 million people globally suffer from depression [1]. In China, depression is a common mental disorder, and about 6% to 8% of total personal medical expenditures were spent on depression and depressive symptoms in 2012 [2]. The cause of depression is complicated, and existing studies attribute depression to a complex interaction of social [3,4] and biological factors [5,6]. Among these factors, the literature has provided evidence that energy poverty is correlated with mental health [7,8,9,10], and few studies have suggested that energy poverty may affect depression [11,12]. However, the evidence of how energy poverty affects depression is still limited, especially in developing countries [10], and no research has focused on rural individuals in developing countries. In addition, none of previous studies have considered the heterogeneous effects of energy poverty on depression across individuals with different levels of depressive symptoms. This study aims at filling these gaps of knowledge.

The concept of energy poverty was introduced by Bradshaw and Hutton [13], and energy poverty can be defined as a lack of access to energy services that are sufficient, reliable, and modern [14]. At present, the definitions of energy poverty can be divided into two major categories [15], which include the difficulty to access modern energy [16,17] and inability to afford adequate energy products and services [18,19,20]. The former definition is primarily applied in developing countries, while the later one is mainly used in developed economies. However, there is no unified definition for energy poverty due to the complicated energy consumption patterns in different regions [15]. For example, some studies define energy poverty as a situation in which a household cannot afford to heat or cool its home to an ambient temperature [10,21]; some define it as a situation that a household’s energy expenditure is over a threshold percentage of income [22,23]; others define it as a situation of low household income and relative high energy costs [19], which has been adopted widely in the most recent literature [12,15,20].

Energy poverty occurs not only in many developing countries, but also in some developed economies. In Europe, energy poverty is a major social problem due to rising energy prices, economic hardship, inequalities and climate variability [24]. It is estimated that 12% households in England [25], 11% to 15% households in Spain [26], 2% to 5% households in Australia [20], and 10% in France [27] are affected. As the largest developing economy in the world, China has had nearly 100% electricity access after 2013 [28]. However, due to large economic disparities in urban and rural regions, household energy poverty status is quite different across the country. For studies in China, previous research has suggested that about 13–19% Chinese households are energy poor using the energy poverty definition of inability to afford [12,15], but studies specifically focusing on rural households are relative limited.

There is a consensus that energy consumption influences health outcomes, and compared with households who are not energy poor, energy-poor households tend to suffer from more adverse health problems [26,29,30]. Sufficient household energy consumption leads to an increased standard of living through home lighting, cooking, space heating, water heating, home cooling, and other home appliances. However, insufficient energy consumption or energy poverty can lead to high indoor temperatures in summer, low indoor temperatures in winter, limited access to home appliances, and indoor air pollution via combustion of wood and coal [31,32]. A large body of literature supports the fact that energy poverty is associated with inferior health outcomes [10,12]. In particular, existing studies suggest that poor thermal and humidity conditions are associated with higher winter mortality and morbidity rates in terms of respiratory and cardiovascular diseases [33,34,35,36,37].

In recent years, there has been a growing body of literature on the relationship between mental health and energy poverty, but most of these studies focus on households in developed economies [7,8,9,10], and few are in developing countries [11,12]. Among these studies, Thomson et al. [7] investigated the relationship between energy poverty and mental well-being across 32 European countries, and their findings suggested that there is a higher incidence of poor mental health amongst energy-poor households in most European countries. Rodriguez-Alvarez et al. [9] found a significant negative association between energy poverty and mental well-being in Spain, suggesting energy-poor individuals are more likely to have poorer mental health compared with their counterparts who are not. With 13 waves of household data in Australia, Awaworyi-Churchill et al. [10] found energy poverty has a negative effect on an individual’s subjective well-being and suggested their general conclusion is robust to alternative energy poverty measures. 

The evidence on how energy poverty affects mental health remains scarce, especially in developing countries [10,12]. For studies in developing countries, Lin and Okyere [11] initiated the first study of its kind in a sub-Saharan African nation and found that energy poverty increases the odds of being mildly, moderately and severely depressed by 0.56, 1.49 and 1.87, respectively, among individuals in Ghana. Regarding studies in China, the study by Nie et al. [12] is among the very first to empirically examine the effect of energy poverty on depression, and they found strong positive effects of energy poverty on depression among Chinese individuals. However, their focus in on the general population, so there is still a gap of knowledge on how energy poverty impacts rural households in China. 

Although previous empirical studies on the relationship between energy poverty and mental health have reached consistent results, their estimated effects of energy poverty on mental health are assumed to be constant among individuals, and no studies have considered the heterogeneous effects of energy poverty on depression across individuals with different levels of depression. Assuming a constant effect of energy poverty on depression for all individuals may yield significant estimation bias and incorrect results. For example, previous studies of Zhang and Yen [38] have pointed out that the effects of physical activity participation on depression are heterogeneous across individuals with different levels of depressive symptoms. They found that physical activity is effective in ameliorating depression among mild-to-moderately depressed individuals but not among severely depressed individuals. Motivated by their study on depression, our study aims to fill this gap in knowledge by investigating the heterogeneous effects of energy poverty on depression across individuals with different levels of depressive symptoms.

In addition to energy poverty, many previous studies suggest that socio-demographic factors of age [39,40], gender [38,40,41,42,43], income [44,45], education status [38,46], marriage status [47], religious beliefs [48], and employment status [49,50] can affect depression. Age is found to be an important factor; for example, Wade and Cairney [40] find a steady decline across age groups after other socio-demographic factors are controlled for, using Canadian samples. Gender is generally accepted as another important factor, and most previous studies have reached consistent results that females are more likely to be depressed than males [37,43,51]. In terms of income, Whooley et al. [44] find low-income young adults more likely to have depressive symptoms than their high-income counterparts, and Zimmerman and Katon [45] suggest a negative association between income and depression symptoms with Kernel regression for both males and females. Considering education and employment status, existing studies suggest less educated and unemployed individuals have a higher risk of being depressed [38,49]. For marriage status, Jang et al. [47] find that separated or widowed Koreans have higher depression scores than their married counterparts.

In this study, we empirically investigate the impact of energy poverty on depression among rural Chinese individuals. Our study contributes to the literature of its kind in several important ways. First, unlike previous studies assuming that the impact of energy poverty on depression is constant across all individuals, we allow such an impact to be varying across individuals with different levels of depression by applying quantile regression approaches. Second, we fill the gap in the knowledge in this field in rural China. To the best of our knowledge, only two recent studies have investigated the impact of energy poverty on well-being among Chinese individuals [12,51], but none of them focused on rural individuals who are more likely to suffer from energy poverty. The remainder of this article is organized as follows: Section 2 introduces the data, measures, and methods, Section 3 reports and interprets the results, Section 4 discusses the results and findings, and Section 5 concludes the article. 

## 2. Materials and Methods

### 2.1. Data and Participants

The research data of this study are drawn from the China Family Panel Studies (CFPS) survey collected and administered by the Institute of Social Science Survey, Peking University. The CFPS is a large national representative dataset which is designed to collect individual, household, and community-level data on socio-demographics, well-being, health, labor, and migration. It covers 25 provinces, municipalities, and autonomous regions in China and constitutes a nationally representative sample that captures both the socio-economic development and the noneconomic well-being of Chinese households [52]. In this study, we use the latest 2018 CFPS data and restrict the study samples to rural households that report non-zero energy expenditures only in 2018. In each rural household, we restrict our samples to individuals who are legal to work, namely at least 16 years old. After removing observations with missing variables and abnormal values, our final sample consists of 13,784 individuals aged at least 16 from 6103 rural households.

### 2.2. The Measure for Depression

**Figure 1:**
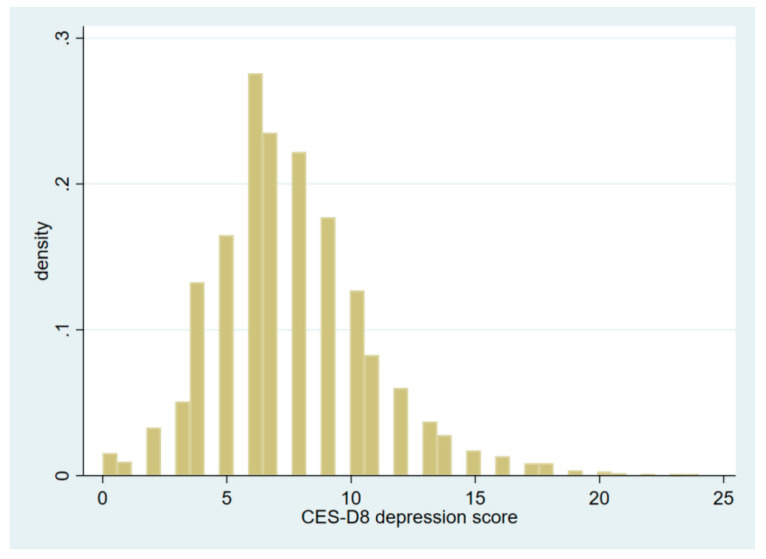
The distributions of CES-D8 depression scores among rural Chinese individuals.

The advantage of the CFPS data is that it includes a questionnaire module of the Center for Epidemiologic Studies Depression Scale (CES-D), which was designed for use in epidemiology studies to assess degrees of depressive symptoms and detect at-risk individuals for depression in the general population [53]. Unlike the traditional 20-item CES-D scale, the 2018 CFPS data use an 8-item CES-D scale to measure depression. Although the 8-item CES-D scale is a short version for the 20-item scale, many studies have suggested that the shorter 8-item CES-D scale is a valid and reliable measure of depressive symptoms [54,55,56,57]. The 8-item CES-D scale includes 8 questions regarding individuals’ recent psychological status; each question corresponds to a score from 0 to 3 based on the individuals’ responses. Therefore, the overall depression score calculated from the CES-D8 scale ranges from 0 to 24. In this study, the 8-item CES-D scale is used to calculate individuals’ depression scores, and the items building up the CES-D8 scale are reported in Table A1 of the Appendix A. The distribution of depression scores among the 13,784 rural individuals is presented in Figure 1.

### 2.3. The Measure for Energy Poverty

There is no unified definition for energy poverty, and researchers define it in several ways. In this study, we follow the most recent studies by adopting Hills [11]’s concept of a “low income high cost” (LIHC) measure, which combines residual income below the income threshold with energy costs above the social average [10,12,15,20]. This measure takes into account the low-income status of households and the high costs of fuel or energy [10]. In particular, similarly to the existing literature [12,15], a household is defined as energy poor if its annual per capita energy cost is above the provincial median while its per capita residual income is below the provincial median, where the residual income is defined as the household’s disposable income minus energy costs and housing costs [15]. In addition to the LIHC measure on which this study primarily focuses, an alternative measure of the “10% indicator” is also used to implement robustness checks. Regarding to the “10% indicator”, a household is defined as energy poor if its energy cost is above 10% of its disposable income [58]. In the final sample, 1141 (18.7%) and 1164 (19.1%) rural households are classified as energy poor under the LIHC and “10% indicator” measure, respectively.

### 2.4. Socio-Demographic Factors

In addition to energy poverty, other factors that may affect individuals’ depression are also included. Following previous studies on depression [12,38,51], we include individual socio-demographic characteristics of age, gender, household size, household income, education, religious beliefs, marriage status, employment status, and location of residence in the model. Summary statistics of depression score, energy poverty measure, and socio-demographic variables are listed in Table 1.

### 2.5. Methods

#### 2.5.1. Ordinary Least Square (OLS) Estimation

For a baseline analysis, we use a standard linear regression, in particular, the OLS regression, to analyze the impact of energy poverty on depression. The OLS regression equation is specified as follows:

(1) $$y_{i}={\mathrm{EP}}_{i}\alpha +x_{i}^{'}\beta +\varepsilon_{i}$$

where $y_{I}$ denotes individual i’s depression scores from the CES-D8 criterion, while ${\mathrm{EP}}_{i}$ is a binary variable indicating whether individual i’s household is energy poor or not. $x_{i}$ is a vector of individual i’s characteristics, such as age, gender, education status, marriage status, employment status, religious beliefs, and fixed effects of location of residence. $\varepsilon_{i}$ is an error term which contains other factors that are unobservable to researchers. α is the coefficient estimate which captures the impact of energy poverty on depression, while β is a vector of coefficient estimate for explanatory variables included in $x_{i}$.

#### 2.5.2. Quantile Regression

The standard linear regression specified above only captures the average relationship between energy poverty and depression scores, and it implicitly assumes that the impact of energy poverty on depression is the same across all individuals in the sample. However, previous studies have found that such an impact on depression can be quite different for individuals with different depressive symptoms [43]. Therefore, the average impact estimated from the OLS regression is far from sufficient to uncover the varying impacts of energy poverty on depression across individuals with a wide range of depression scores. In this section, we apply the quantile regression approach developed by Koenker and Bassett [59] to investigate the varying impacts of energy poverty on depression of individuals in rural China.

The quantile regression has several advantages over the standard linear regression. First, the quantile regression provides a complete description of the relationship between the dependent variable and a set of explanatory variables at different percentiles, for example, the 25th and 75th percentiles of the dependent variable. Second, the point estimates of the quantile regression are robust to sample outliers, heteroscedasticity, and skewness on the dependent variables [60,61]. According to Koenker and Bassett [59], the quantile regression of depression score $y_{i}$ given a set of explanatory variables $x_{i}$ is specified as follows:

(2) $$Q_{\tau }\left(y_{i}|x_{i}\right)=x_{i}^{'}\beta_{\tau }$$

where $Q_{\tau }\left(y_{i}|x_{i}\right)$ denotes a conditional quantile function of the th conditional quantile of depression score $y_{i}$ and $\beta_{\tau }$ is a vector of regression coefficients in the quantile regression. The regression coefficients are estimated by minimizing the sum of absolute values of error terms [59,60,62]. Therefore, the τth quantile regression coefficient $\beta_{\tau }$ can be estimated by minimizing

(3) $$Q\left(\beta_{\tau }\right)=\sum_{i:y_{i}\geq x_{i}^{'}\beta }^{N}\tau \left|y_{i}-x_{i}^{'}\beta_{\tau }\right|+\sum_{i:y_{i}<x_{i}^{'}\beta }^{N}\left(1-\tau \right)\left|y_{i}-x_{i}^{'}\beta_{\tau }\right|$$

The non-differentiable function defined above can be minimized via the simplex method, which is guaranteed to yield a solution. Standard errors are calculated with the bootstrap method. 

In order to investigate the impact of energy poverty on depression scores of individuals in rural China, the quantile regression framework of Equation (1) can be now expressed as follows:

(4) $$y_{i}={\mathrm{EP}}_{i}\alpha_{\tau }+x_{i}^{'}\beta_{\tau }+\varepsilon_{i}$$

where i=1,…,N denotes the th individual in the sample and τ=0.1, 0.25, 0.5, 0.75, 0.9 is the quantile being analyzed in this article. 

#### 2.5.3. Quantile Regression with Instrumental Variables

The use of OLS and quantile regression creates a potential endogeneity problem in energy poverty, which includes the possibility of omitted variable bias, measurement error, and simultaneity bias [12]. The endogeneity problem occurs when the unobserved variable or omitted variable in the error term is correlated with explanatory variables in the regression, for example, $E\left(\;x\;\text{′}\;\varepsilon \right)\neq 0$. In our case, it has been argued that a higher level of social support is linked to better subjective well-being [63] as well as a lower likelihood of energy poverty [12]; thus, social support as an omitted variable may lead to underestimation of the impact of energy poverty [20]. Considering the effect of energy poverty on depression, another potential source of endogeneity arises from the systematic measurement error in calculating the measure of energy poverty [10].

To address the above endogeneity problems in our estimation, we follow existing literature by using provincial level electricity and natural gas prices as instrumental variables to instrument the measure of energy poverty under the assumption that energy prices increase with energy expenditures and thus the possibility of being energy poor [10,12,64]. 

Unlike quantile regression, the estimation algorithm for instrumental variable (IV) quantile regression is much more difficult and complicated. Existing estimation methods for IV quantile regression mainly include the Instrumental Quantile Treatment Effect (IVQR) model [65,66], Method of Moments—Quantile Regression [67], and the Generalized Quantile Regression (GQR) method [68]. The GQR method is based on the framework of Chernozhukov and Hansen [65] but extends to permit multiple endogenous variables which can be either discrete or continuous. More importantly, the GQR is simple to implement and fast to converge with standard statistical software. Due to these advantages of the GQR method, in this study, we will apply the GQR method to estimate the quantile regression equation defined above with energy prices as instrumental variables.

## 3. Results

### 3.1. The OLS Estimation Results

As discussed earlier, our primary measure for energy poverty is Hills [19]’s “low income high cost” measure [10,12,20]. Thus, the remaining analyses of this article are based on the results of this energy poverty measure (EP1). Column 2 of Table 2 presents the OLS regression estimation results with EP1 as the energy poverty measure. The coefficient estimate of EP1 is 0.158, which is positive and significant at the 5% level of significance, suggesting that energy poverty is positively associated with individuals’ depression scores on average. In addition to household energy poverty status, individuals’ socio-demographic characteristics and variables of age, household size, gender, religious beliefs, marriage status, employment status, and location of residence are found to play roles in affecting depression. Age is found to be positively associated with individuals’ depression scores. In particular, all else equal, a 10-year increase in age would increase one’s depression scores by 0.246 points on average. The impact of household size on depression is significant but the magnitude is small. As expected, gender plays a vital role in affecting one’s depression scores, and males have 0.730 points lower depression scores than females on average, suggesting females are more likely to be depressed. This finding is also consistent with previous studies [38,69]. Compared to an individual who has no religious beliefs, an individual with religious beliefs has 0.438 points higher depression scores on average. For marriage status, both single and married individuals have lower depressions scores compared to those who are widowed. Specifically, all else equal, married individuals have the lowest depression scores on average, suggesting social support emanating from spouses reduces the risk of depression [70]. In contrast to unemployed individuals, individuals who are employed have lower depression scores on average. Moreover, location of residence plays a role in determining individuals’ depression scores. For example, compared with individuals living in the central part of rural China, individuals living in the north part of rural China have 0.385 points lower depression scores on average.

### 3.2. Quantile Regression Estimation Results

Following Koenker and Hallock [60], a quantile regression on depression scores is estimated based on the 10th, 25th, 50th, 75th, and 90th quantiles. Columns of 3–7 in Table 2 present the estimation results of a quantile regression with EP1 as the energy poverty measure. Results show that quantile estimates of energy poverty (EP1) at the lower (10th and 25th) and middle (50th) quantiles of depression score have small and insignificant impacts on depression. In particular, we cannot reject the null hypothesis that the quantile estimates of EP1 at the lower and middle quantile are equal to zero. This finding suggests that energy poverty has no impact on depression for individuals at the lower and middle quantiles. However, results show that quantile estimates of energy poverty at the upper (75th and 90th) quantiles of depression score have significant positive impacts on depression. In particular, the impact sizes are 0.351 and 0.542 at the 75th and 90th quantiles, respectively, and both are much larger than the impact size (0.158) of EP1 on depression from the OLS regression. 

**Figure 2:**
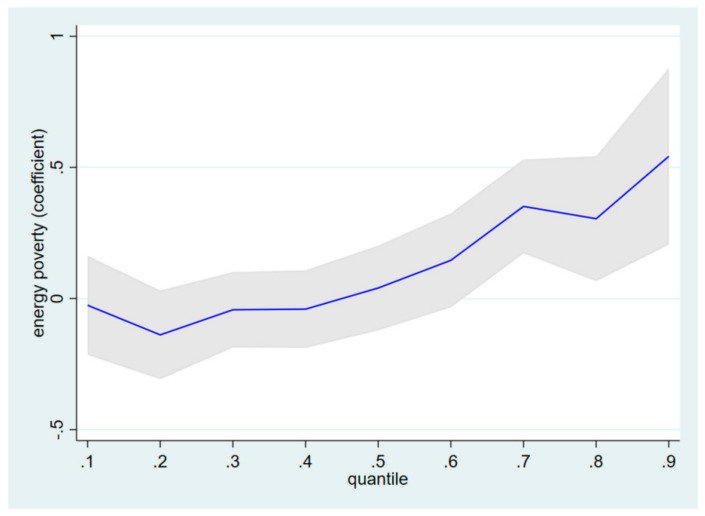
Quantile regression estimates for the impact of energy poverty (EP1) on depression. Notes: The *x*-axis denotes the 9 conditional quantiles of the CES-D8 depression scores, and the *y*-axis presents the coefficient estimates of energy poverty measure (EP1). Shaded areas correspond to 95% confidence intervals of quantile estimation.

Figure 2 displays the relationship between nine conditional quantiles (from the 10th to the 90th) of depression scores and their corresponding EP1 coefficient estimates. It is clear to identify that the EP1 coefficient estimates are near zero at the lower and middle quantiles, and strictly positive at the upper quantiles. Moreover, an upward trend of EP1 coefficient after the middle quantile is observed. Since the OLS coefficient estimate reflects the average impact of energy poverty on depression across all individuals, interpreting results based on OLS coefficient estimates would overestimate (underestimate) the impacts of energy poverty on depression at the lower (upper) quantile. 

For the coefficient estimates of socio-demographic characteristics, quantile regression also yields more reliable and richer results. For example, referring to Table 2, the coefficient of age is positive and significant at the 1% level of significance at all five quantiles of depression scores. However, the estimated coefficient of age is smaller at the 10th quantile (0.195) and larger at the 90th quantile (0.332) than that of the OLS-estimated coefficient (0.246), suggesting the effects of age on depression are heterogeneous across individuals with different depression scores. Household size has no effect on depression for individuals at the 10th quantile. In particular, we find that the absolute value of male coefficient estimate is smaller at the lower quantile and larger at the upper quantile than that of the OLS coefficient estimate. For the interpretation, males are found to yield 0.480, 0.650, 0.758, 0.720, and 0.969 lower depression scores than females at the 10th, 25th, 50th, 75th, and 90th quantiles, respectively. Moreover, variables of education status, religious belief, marriage status, employment status, and location of residence are also found to affect individuals’ depression scores at different conditional quantiles, and coefficient estimates can be interpreted similarly. 

### 3.3. Instrumental Variable Quantile Regression Estimation Results

Although the quantile regression yields more realistic and richer results than OLS regression, the endogeneity issue in energy poverty may still bias the coefficient estimates. In order to avoid the potential endogeneity problem of energy poverty, we apply the GQR method developed by Powell [68] to estimate the quantile regression with instrumental variables (IV).

Table 3 presents the estimation results of an IV quantile regression with EP1 as the energy poverty measure. Results show that IV quantile estimates of energy poverty (EP1) at the lower and middle quantiles of depression score have small and insignificant impacts on depression, which are consistent with results from the regular quantile regression discussed earlier. However, at the 75th and 90th quantiles of depression scores, the IV quantile regression yields stronger effects on energy poverty compared to the regular quantile regression. These results suggest that ignoring the endogeneity issue would underestimate the impact of energy poverty on depression at upper quantiles. Specially, the IV quantile estimates denote that energy poor individuals have 0.446 and 0.573 higher depression scores, respectively, than their non-poor counterparts at the 75th and 90th quantiles of depression scores.

**Figure 3:**
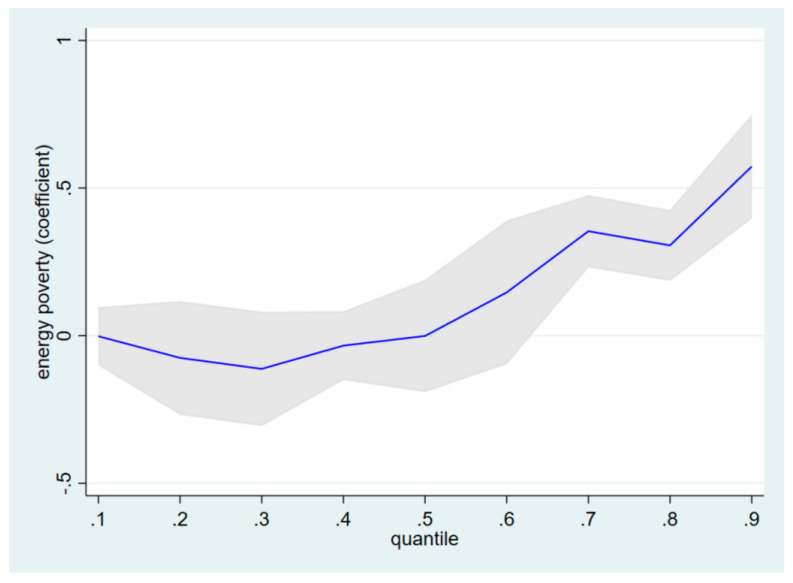
Instrumental variable quantile regression estimates for the impact of energy poverty (EP1) on depression. Notes: The *x*-axis denotes the nine conditional quantiles of the CES-D8 depression scores, and the *y*-axis presents the coefficient estimates of energy poverty measure (EP1). Shaded areas correspond to 95% confidence intervals of quantile estimation.

Figure 3 displays the relationship between nine conditional quantiles of depression scores and their corresponding EP1 coefficient estimates from the IV quantile regression. The overall trend of the EP1 coefficient along the *x*-axis is very similar to that of Figure 2. However, we find the EP1 coefficient estimates from the IV quantile regression yield narrower confidence intervals at the upper quantiles compared to those from the regular quantile regression, suggesting the IV quantile regression generates more reliable and robust estimates than the regular quantile regression. In addition, Figure 3 confirms that energy poverty has no effect on depression for individuals at the lower to middle quantiles of depression scores. 

Considering the coefficient estimates of socio-demographic characteristics, the IV quantile regression yields smaller standard deviations of coefficient estimates than the regular quantile regression. For example, referring to Table 3, the coefficient of household income is significant at the 1% level of significance at the 10th, 50th, and 90th quantiles, which are not significant with the regular quantile regression. Though the effects are small, the negative association between income and depression at the middle and upper quantiles reveals that the increase in income would reduce depression for those with moderate to severe symptoms, which is consistent with existing studies [38,51]. Except the standard deviations of coefficient estimates, the coefficient estimates of socio-demographic variables from the IV quantile regression are close to those from the regular quantile regression. For example, the IV quantile estimates suggest that compared to female individuals, male individuals have 0.476, 0.648, 0.755, 0.712, and 0.982 lower depression scores at the 10th, 25th, 50th, 75th, and 90th quantiles, respectively, whose impact sizes are close to those from the regular quantile regression.

Some coefficient estimates of socio-demographic variables are significant under the IV quantile regression, while they are not under the regular quantile regression. For example, referring to education status, Table 3 shows that compared to individuals who have bachelor’s degrees, individuals who have only a primary school education or lower have 0.367 and 0.207 (0.591 and 1.487) lower (higher) depression scores at the 10th and 25th (75th and 90th) quantiles of depression scores. This finding suggests less educated individuals at lower quantiles are less likely to be depressed, while they are more likely to be depressed at upper quantiles of depression scores compared to more educated individuals. In terms of employment status, employed individuals have 0.109, 0.188, 0.144, and 0.177 lower depression scores at each corresponding quantile of depression scores compared to their counterparts who are unemployed. This finding that unemployment is associated with higher risks of depression is consistent with previous studies [38,49].

## 4. Discussion

### 4.1. Robustness Checks to Alternative Measures

In this study, our primary analyses are based on Hills [11]’s concept of a “low income high cost” (LIHC) measure for energy poverty; therefore, results and findings can change with different measures. In order to verify that our estimation results are robust and not sensitive to different energy poverty measures, we conduct robustness checks by using the alternative 10% energy expenditure measure to re-estimate the OLS, quantile regression, and IV quantile regression models. Corresponding estimation results are reported in the Appendix A. 

**Figure A1:**
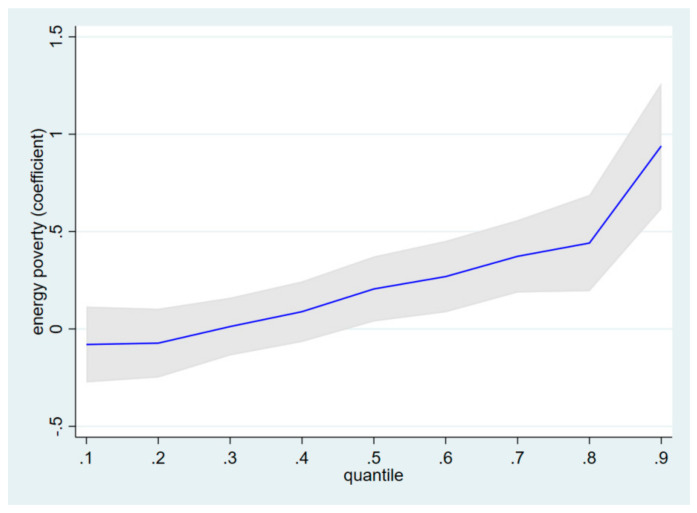
Quantile regression estimates for the impact of energy poverty (EP2) on depression. Notes: the *x*-axis denotes the 9 conditional quantiles of the CES-D8 depression scores, and the *y*-axis presents the coefficient estimates of energy poverty measure (EP2). Shaded areas correspond to 95% confidence intervals of quantile estimation.

In column 2 of Table A2 in the Appendix A, the OLS regression with an alternative energy poverty measure (EP2) yields a positive and significant coefficient estimate, consistent with the results of the primary measure. In particular, the coefficient estimate of EP2 is 0.244, which is slightly larger in magnitude than our primary measure EP1. In terms of socio-demographic factors, coefficient estimates are quite similar from regressions with EP1 and EP2 measures, suggesting that the estimation results are robust and not sensitive to different energy poverty measures. Columns 3–7 of Table A2 in the Appendix A present estimation results of the quantile regression model with the EP2 measure. As with our coefficient estimate of EP1, the coefficient estimate of EP2 is not significant in affecting individuals’ depression scores at the 10th and 25th quantiles, while it is significant in affecting depression scores at the 50th, 75th and 90th quantiles. Although the effect of energy poverty is not significant at the middle quantile when using EP1 as the measure, its impacts on depression are consistent with those of the EP2 measure at other quantiles. Figure A1 in the Appendix A presents the relationship between nine conditional quantiles of depression scores and their corresponding EP2 coefficient estimates. Similar to Figure 2, an upward trend of the EP2 coefficient is observed, and estimates are near zero at lower quantiles while strictly positive at upper quantiles. For individual socio-demographic characteristics, coefficient estimates are similar to quantile regressions with EP1 and EP2 measures. The robustness checks suggest the alternative measure of energy poverty does not change our primary findings with OLS and quantile regression models. 

**Figure A2:**
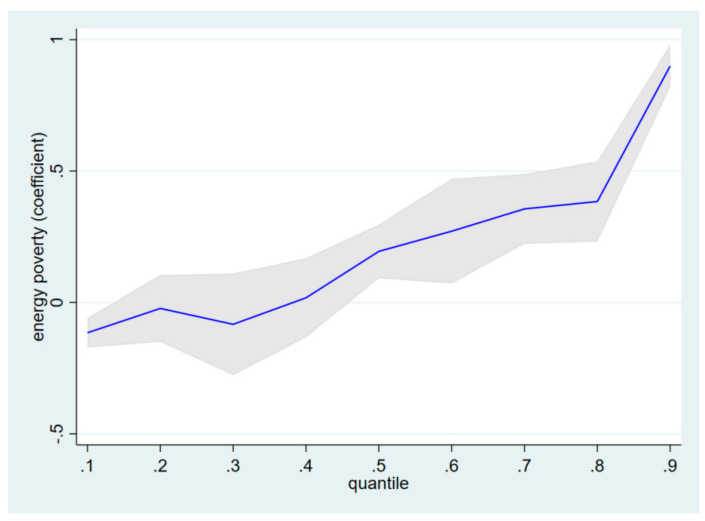
Instrumental variable quantile regression estimates for the impact of energy poverty (EP2) on depression. Notes: the *x*-axis denotes the 9 conditional quantiles of the CES-D8 depression scores, and the *y*-axis presents the coefficient estimates of energy poverty measure (EP2). Shaded areas correspond to 95% confidence intervals of quantile estimation.

For the IV quantile regression model, Table A3 in the Appendix A presents the results with the alternative EP2 measure. As expected, we find significant and positive impacts of energy poverty on depression at the upper quantiles with the alternative EP2 measure, whose results are consistent with our primary EP1 measure. Although the coefficient estimate of EP2 is negative and significant at the 10th quantile of depression scores, its magnitude is small and would not change our main finding that energy poverty increases individuals’ depression scores at the upper quantiles. In addition, Figure A2 presents the relationship between nine conditional quantiles of depression scores and their corresponding EP2 coefficient estimates from the IV quantile regression. Similar to Figure 3, we observe an overall upward trend of the EP2 coefficient along the *x*-axis, but the curve of EP2 coefficient is steeper than that of the EP1 coefficient at upper quantiles of depression scores, suggesting the impact of EP2 is larger than that of EP1 at upper quantiles. For coefficient estimates of socio-demographic variables, IV quantile regressions with EP1 and EP2 energy poverty measures do not generate significantly different results. Therefore, the robustness checks suggest that the estimation results are robust and the alternative measure of energy poverty does not change our primary findings. 

### 4.2. Theoretical Implications

Most previous studies rely exclusively on linear regressions to investigate the impact of energy poverty on health status [10,12], which implicitly assumes that the magnitude of the energy poverty coefficient estimate is constant across individuals with different health statuses. However, as suggested by Zhang and Yen [38], such impacts on depression may vary across individuals with different risks of depression. Our empirical results from both quantile regression and IV quantile regression models provide strong evidence that the impact of energy poverty on depression varies across individuals with different depression risks. Thus, quantile regression is preferred to OLS regression in interpreting the impact of energy poverty on depression. Specifically, for a quantile regression, each point estimate of energy poverty can be interpreted as the impact of being energy poor on an individual’s depression score, holding other variables fixed. In our case, individuals at the upper quantile who are energy poor have higher depression scores than their counterparts who are not energy poor. In addition, our results suggest that the impact of energy poverty on depression is larger among individuals with severe depressive symptoms. 

As suggested by the existing literature [10,12,64], the energy poverty measure is potentially endogenous since it is likely to be correlated with unobservable characteristics in the error terms of the estimating regression. Without dealing with such endogeneity issues, the estimation results would be biased. By using an IV quantile regression model, we are able to address the potential endogeneity in energy poverty measure and correctly estimate corresponding parameters in the quantile regression model. In our case, although findings from both quantile regression and IV quantile regression models are consistent, we find that the estimated parameter coefficients are slightly different. For example, with the EP1 measure, the coefficient estimate of energy poverty is 0.351 (0.542) and 0.446 (0.573) at the 75th (90th) quantile of depression scores for quantile and IV quantile regression, respectively. These results suggest that with the EP1 measure, the estimated impact of energy poverty on depression is smaller without considering potential endogeneity issues. In the context of depression, the potential endogeneity of energy poverty may be caused by (i) the omitted variables that affect depression but are not included in the regression, for instance, individuals’ previous depression records; (ii) the measurement error of energy poverty. For instance, respondents may not accurately answer their energy costs which may lead to measurement error; (iii) simultaneity bias, which suggests that energy poverty affect depression, but depression may also affect energy poverty status simultaneously. For instance, depressed individuals may yield lower cognitive ability which makes them more likely to misreport energy costs. For empirical studies, correctly estimating parameters is the cornerstone of analyses. With regard to the above sources of endogeneity in energy poverty, the use of an IV quantile regression model can reduce the estimation bias and yield reliable estimates.

### 4.3. Policy Implications

In 2015, the United Nations set some sustainable development goals (SDG) aiming to end poverty in all forms and promote healthy lives and well-being for all individuals worldwide by 2030. For developing countries which had eradicated absolute poverty, reducing energy poverty and promoting general health are some of the major challenges under the SDG. Although China has achieved great economic prosperity in recent decades and declared its success in eliminating absolute poverty in 2020, about 36% of its population still lives in less developed, rural areas with restricted access to modern energy and inadequate healthcare services. In our study, we find about 16–17% rural households in China are classified as energy poor under the EP1 and EP2 measures, which is higher than Australia’s 3–5% [20], Spain’s 11% to 15% [26], and France’s 10% [27], but lower than Pakistan’s 55% [71], Ghana’s 81% [72], Uganda’s 66% [73], and Sri Lanka’s 72% [74]. Therefore, reducing energy poverty is still important in rural China to increase residents’ welfares. Energy poverty is found to yield negative health effects [26,29,30], and the primary purpose of this study is to explore the impact of energy poverty on depression among rural Chinese individuals. We find strong evidence that energy poverty increases the depression scores of individuals at the middle to upper quantiles of depression, and the impacts of energy poverty on depression are larger for individuals with higher risks. Our findings are critical to the sustainable development of poor and emerging economies and can assist policy makers in achieving the SDG target of alleviating energy poverty and promoting general health.

To reduce the risks of depression in rural areas, one channel is through the alleviation of energy poverty. To do that, we propose several policy recommendations based on our findings. First, the local government should increase investment in energy facilities, such as power grid upgradation, gas pipeline construction, and energy equipment maintenance in less developed rural areas to improve residents’ accessibility to modern energy. Secondly, the government should set up a standardized procedure to identify and record energy-poor households based on local households’ energy consumption patterns and habits. Third, for households who are identified as energy poor, the government should subsidize their use of energy. The subsidy to energy-poor households can either be in the form of a price subsidy or a quantity subsidy. 

In addition to energy poverty, we find socio-demographic variables can play critical roles in affecting depression. In particular, we find age, household size, gender, education status, religious beliefs, marriage status, employment status, and location of residence play some roles in affecting depression. Our findings from the IV quantile regression suggests individuals who are aged, females, widowed, and unemployed and who have limited education, a smaller household size, and hold religious beliefs have higher risks of being depressed, and the impacts of these socio-demographic factors on depression are larger among individuals at the upper quantiles of depression. These findings suggest policy makers and clinical practitioners should pay particular attention to the above groups of individuals who have higher risks of depression. 

### 4.4. Limitations

While we have made several contributions to the existing literature, limitations remain in this study. Firstly, energy poverty measures are calculated from individuals’ self-reported answers to energy costs rather than their actual consumption. Measures based on self-reported questions may yield reporting errors and eventually affect research results. Secondly, objective energy poverty measures may not reflect households’ actual energy poverty status. Due to the data limitation, we do not observe households’ self-reported energy poverty status. Solely relying on measures calculated from energy costs may mismatch households’ energy poverty status. For example, households who use woods and biomass are actually energy poor, but they may be excluded from the energy-poor category due to low energy costs. Thirdly, the study is restricted to regional populations in rural China. With data from only one country, the heterogeneous effects of energy poverty on depression cannot be explicitly uncovered. 

## 5. Conclusions

There exist many studies on the impact of energy poverty on health-related outcomes, but there is a dearth of studies focusing on depression, especially among rural households. In this study, we empirically investigate the impact of energy poverty on depression among individuals in rural China and attempt to fill this gap of knowledge in the empirical literature. The most recent individual and household-level data from the CFPS are used and empirical analyses are carried out by estimating quantile regression models. 

By considering the potential endogeneity in energy poverty measures, our estimates from the IV quantile regression suggest a strong positive impact of energy poverty on depression at the upper quantile of depression scores, but no impact at the middle and lower quantiles. In contrast to existing studies which concentrate exclusively on the average impact by assuming energy poverty affects all individuals identically [10,12], our quantile regression model allows heterogeneous impacts of energy poverty on individuals with different depression scores. In particular, we find the linear regression with constant energy poverty coefficient estimate will significantly overestimate the impact of energy poverty on depression for individuals with low depression scores. With results from the IV quantile regression, we find that energy poverty does not affect the depression of low-risk individuals (with low depression scores), but it does affect that of high-risk individuals (with high depression scores). In addition, the robustness checks with alternative energy poverty measures suggest our estimation results are robust and our primary findings are reliable.

We find individuals’ socio-demographic factors play differential roles in affecting depression at different quantiles of depression scores. Age is positively associated with the risk of depression and its impact is larger among individuals with higher risks. Household size has been found to negatively associate with depression risks and income has a trivial effect on depression. Consistently with previous studies [38,51], we find males are less likely to be depressed than females. Rural individuals’ education status, religious beliefs, marriage status, employment status, and location of residence also play some roles in affecting his or her current depression. 

This study is among the first to evaluate the impact of energy poverty on depression among rural individuals in China with quantile regression models. Our novel finding that energy poverty affects the depression scores of high-risk individuals but not low-risk individuals suggests that policy makers should take energy poverty into consideration when deliberating health-related policies. Since energy poverty has been found to increase depression scores among high-risk individuals, an energy price subsidy may serve as a useful policy instrument for energy-poor individuals to lower their energy expenditure and reduce depressive symptoms. Future studies should use alternative depression measures to verify our findings and if possible, investigate and compare the long-term effects of energy poverty on mental health between urban and rural individuals. 

## Figures and Tables

**Table 1:** Variable Definitions and Sample Statistics.

Variables	Definition	Mean
*Depression measure*		
CES-D8	The CES-D8 depression score, ranging from 0–24	7.610
		(3.226)
*Energy poverty measures*		
EP1	The “Low income high cost” (LIHC) measure	0.166
EP2	The “10% indicator” measure	0.164
*Continuous explanatory variables*		
Age	Respondent age in years (≥16)	47.599
		(16.611)
Household size	Respondent’s household size	4.491
		(2.109)
Household income	Respondent’s household income in thousand yuan	69.235
		(84.761)
*Binary explanatory variables*		
Male	Respondent is male	0.501
Primary School	Respondent’s maximum education is primary school or below	0.536
Middle to high school	Respondent’s maximum education is middle to high school	0.414
Some college	Respondent has some college but not a bachelor’s degree	0.032
Bachelor’s degree	Respondent has a Bachelor’s degree or above (reference)	0.018
Religious belief	Respondent has religious beliefs	0.413
Single	Respondent is single, separate, or unmarried couple	0.124
Married	Respondent is married	0.799
Divorced	Respondent is divorced	0.016
Widowed	Respondent is widowed (reference)	0.060
Employed	Respondent is employed	0.774
Northeast	Respondent resides in northeast part of China	0.118
North	Respondent resides in north part of China	0.131
Northwest	Respondent resides in northwest part of China	0.205
South	Respondent resides in south part of China	0.098
Southwest	Respondent resides in southwest part of China	0.156
East	Respondent resides in east part of China	0.141
Central	Respondent resides in central part of China (reference)	0.149
*Instrumental variables*		
Electricity price	The average electricity price of respondent’s province, yuan per kwh	0.525
		(0.039)
Natural gas price	The average natural gas price of respondent’s province, yuan per liter	2.785
		(0.604)
Sample size		13,784

Standard deviations of continuous variables are in parentheses.

**Table 2:** Estimated parameter coefficients from the OLS and quantile regression models with EP1 as energy poverty measure.

	OLS	Quantile Regression				
Variable		q10	q25	Median	q75	q90
Ep1	0.158 ** (0.073)	–0.026 (0.096)	–0.092 (0.082)	0.040 (0.083)	0.351 *** (0.112)	0.542 *** (0.195)
Age	0.246 *** (0.022)	0.195 *** (0.068)	0.186 *** (0.055)	0.191 *** (0.028)	0.238 *** (0.036)	0.332 *** (0.052)
Household size	−0.034 ** (0.014)	0.008 (0.013)	−0.023 * (0.012)	−0.043 *** (0.016)	−0.054 *** (0.017)	−0.056 * (0.031)
Household income	0.000 (0.000)	0.000 (0.000)	0.000 (0.000)	0.000 (0.000)	−0.001 (0.001)	−0.001 (0.001)
Male	−0.730 *** (0.056)	−0.480 *** (0.178)	−0.650 *** (0.103)	−0.758 *** (0.061)	−0.720 *** (0.073)	−0.969 *** (0.128)
Primary School	0.192 (0.212)	−0.328 (0.232)	−0.245 (0.209)	−0.103 (0.183)	0.643 *** (0.219)	1.438 *** (0.386)
Middle to high school	−0.070 (0.207)	−0.196 (0.202)	−0.148 (0.196)	−0.236 (−0.173)	0.234 (0.209)	0.391 (0.351)
Some college	−0.248 (0.249)	−0.217 (0.272)	−0.225 (0.233)	−0.346 * (0.208)	0.002 (0.236)	0.037 (0.446)
Religious belief	0.438 *** (0.055)	0.227 ** (0.098)	0.299 *** (0.091)	0.408 *** (0.063)	0.542 *** (0.074)	0.477 *** (0.131)
Single	−0.570 *** (0.160)	−0.094 (0.364)	−0.330 (0.238)	−0.723 *** (0.209)	−1.015 *** (0.240)	−0.909 *** (0.333)
Married	−0.953 *** (0.120)	−0.431 (0.269)	−0.540 *** (0.175)	−1.012 *** (0.180)	−1.352 *** (0.186)	−1.629 *** (0.256)
Divorced	−0.066 (0.242)	−0.678 (0.654)	0.172 (0.326)	−0.036 (0.303)	−0.227 (0.341)	−0.501 (0.481)
Employed	−0.150 ** (0.069)	−0.114 (0.086)	−0.193 ** (0.093)	−0.137 (0.094)	−0.193 * (0.106)	0.001 (0.156)
Northeast	0.385 *** (0.105)	−0.003 (0.102)	0.181 * (0.107)	0.242 * (0.132)	0.367 ** (0.149)	0.877 *** (0.231)
North	0.838 *** (0.101)	0.477 ** (0.187)	0.729 *** (0.169)	0.847 *** (0.090)	0.793 *** (0.110)	0.982 *** (0.224)
Northwest	0.745 *** (0.091)	0.064 (0.130)	0.418 *** (0.151)	0.846 *** (0.096)	0.946 *** (0.120)	1.045 *** (0.190)
South	0.312 *** (0.110)	0.109 (0.134)	0.190 * (0.103)	0.291 ** (0.117)	0.281 *** (0.112)	0.301 (0.213)
Southwest	0.461 *** (0.097)	0.042 (0.107)	0.276 ** (0.115)	0.458 *** (0.112)	0.463 *** (0.129)	0.758 *** (0.224)
East	−0.073 (0.100)	0.000 (0.104)	0.025 (0.092)	−0.035 (0.106)	−0.120 (0.125)	−0.152 (0.195)
Constant	7.261 *** (0.289)	3.880 *** (0.472)	5.602 *** (0.469)	7.580 *** (0.314)	8.968 *** (0.335)	10.339 *** (0.583)
N	13,784					

Asymptotic standard errors are in parentheses. *** *p* < 0.01, ** *p* < 0.05, * *p* < 0.10.

**Table 3:** Estimated parameter coefficients from the instrumental variable quantile regression model with EP1 as energy poverty measure.

	Quantile Regression				
Variable	q10	q25	Median	q75	q90
Ep1	−0.002 (0.050)	−0.126 (0.123)	−0.001 (0.097)	0.446 *** (0.095)	0.573 *** (0.089)
Age	0.192 *** (0.008)	0.186 *** (0.008)	0.204 *** (0.012)	0.245 *** (0.013)	0.326 *** (0.009)
Household size	0.007 (0.005)	−0.024 *** (0.005)	−0.040 *** (0.009)	−0.046 *** (0.006)	−0.061 *** (0.006)
Household income	0.000 *** (0.000)	0.000 (0.000)	−0.001 *** (0.000)	0.000 * (0.000)	−0.001 *** (0.000)
Male	−0.476 *** (0.022)	−0.648 *** (0.019)	−0.755 *** (0.021)	−0.712 *** (0.030)	−0.982 *** (0.030)
Primary School	−0.367 *** (0.065)	−0.207 *** (0.067)	−0.015 (0.083)	0.591 *** (0.106)	1.487 *** (0.065)
Middle to high school	−0.231 *** (0.060)	−0.113 * (0.067)	−0.132 * (0.079)	0.174 * (0.104)	0.449 *** (0.065)
Some college	−0.265 *** (0.063)	−0.203 *** (0.065)	−0.237 ** (0.098)	−0.041 (0.118)	0.080 (0.091)
Religious belief	0.233 *** (0.014)	0.313 *** (0.021)	0.426 *** (0.022)	0.550 *** (0.026)	0.460 *** (0.028)
Single	−0.074 (0.054)	−0.305 *** (0.068)	−0.678 *** (0.075)	−0.965 *** (0.105)	−0.949 *** (0.055)
Married	−0.421 *** (0.047)	−0.517 *** (0.060)	−1.003 *** (0.070)	−1.338 *** (0.079)	−1.624 *** (0.032)
Divorced	−0.640 *** (0.164)	0.194 * (0.116)	0.006 (0.145)	−0.176 (0.123)	−0.436 *** (0.072)
Employed	−0.109 *** (0.022)	−0.188 *** (0.027)	−0.114 *** (0.035)	−0.177 *** (0.032)	0.000 (0.036)
Northeast	−0.012 (0.023)	0.178 *** (0.034)	0.225 *** (0.058)	0.363 *** (0.057)	0.841 *** (0.052)
North	0.475 *** (0.025)	0.723 *** (0.046)	0.864 *** (0.049)	0.782 *** (0.045)	0.995 *** (0.042)
Northwest	0.069 *** (0.025)	0.426 *** (0.038)	0.846 *** (0.054)	0.932 *** (0.046)	1.033 *** (0.044)
South	0.097 ** (0.037)	0.184 *** (0.035)	0.296 *** (0.060)	0.297 *** (0.045)	0.299 *** (0.046)
Southwest	0.030 (0.027)	0.263 *** (0.037)	0.458 *** (0.050)	0.477 *** (0.045)	0.779 *** (0.049)
East	−0.006 (0.021)	0.020 (0.031)	−0.033 (0.045)	−0.141 *** (0.047)	−0.156 *** (0.038)
Constant	3.916 *** (0.099)	5.550 *** (0.129)	7.385 *** (0.129)	8.928 *** (0.161)	10.370 *** (0.096)
N	13,784				

Asymptotic standard errors are in parentheses. *** *p* < 0.01, ** *p* < 0.05, * *p* < 0.10.

## Data Availability

The data of this research are public available.

## Appendix A

**Table A1:** Items of the CES-D8 Scale.

Over the Last Week, How Often (How Many Days) Have You Been Bothered by Any of the Following Problems?	Not at All (<1 Day)	Sometimes (1–2 Days)	Often (3–4 Days)	Most of the Time (5–7 Days)
I felt depressed	0	1	2	3
I felt everything I did was an effort	0	1	2	3
I slept restlessly	0	1	2	3
I was unhappy	0	1	2	3
I felt lonely	0	1	2	3
I did not enjoy life	0	1	2	3
I felt sad	0	1	2	3
I was unable to get going	0	1	2	3

Note: The depression score calculated from the 8-item scale above ranges from 0 to 24. Higher depression score shows more severe depressive symptoms.

**Table A2:** Estimated parameter coefficients from the OLS and quantile regression models with EP2 as energy poverty measure.

	OLS	Quantile Regression				
Variable		q10	q25	Median	q75	q90
Ep2	0.244 *** (0.076)	–0.800 (0.112)	–0.021 (0.080)	0.206 ** (0.091)	0.398 *** (0.118)	0.940 *** (0.193)
Age	0.241 *** (0.022)	0.201 *** (0.073)	0.187 *** (0.036)	0.189 *** (0.021)	0.231 *** (0.035)	0.336 *** (0.046)
Household size	−0.034 ** (0.014)	0.008 (0.015)	−0.023 ** (0.013)	−0.038 ** (0.015)	−0.050 *** (0.017)	−0.055 ** (0.028)
Household income	−0.000 (0.000)	0.000 (0.000)	0.000 (0.000)	0.000 * (0.000)	−0.000 *** (0.001)	0.000 * (0.001)
Male	−0.730 *** (0.056)	−0.487 ** (0.190)	−0.659 *** (0.073)	−0.764 *** (0.056)	−0.729 (0.076)	−1.025 *** (0.125)
Primary School	0.195 (0.211)	−0.350 (0.276)	−0.264 (0.245)	−0.089 (0.182)	0.551 *** (0.208)	1.296 *** (0.368)
Middle to high school	−0.064 (0.207)	−0.210 (0.255)	−0.162 (0.232)	−0.223 (0.176)	0.134 (0.209)	0.378 (0.322)
Some college	−0.243 (0.249)	−0.252 (0.295)	−0.202 (0.262)	−0.297 (0.229)	−0.047 (0.249)	−0.097 (0.412)
Religious belief	0.436 *** (0.055)	0.244 ** (0.110)	0.301 *** (0.666)	0.399 *** (0.058)	0.520 *** (0.077)	0.503 *** (0.126)
Single	−0.578 (0.160)***	−0.068 (0.429)	−0.315 (0.227)	−0.719 *** (0.205)	−1.010 *** (0.254)	−1.016 *** (0.334)
Married	−0.955 *** (0.120)	−0.429 * (0.308)	−0.521 *** (0.157)	−1.042 *** (0.170)	−1.324 *** (0.183)	−1.744 *** (0.270)
Divorced	−0.067 (0.242)	−0.573 (0.565)	0.154 (0.300)	−0.016 (0.326)	−0.283 (0.432)	−0.482 (0.563)
Employed	−0.144 ** (0.069)	−0.114 (0.083)	−0.184 ** (0.071)	−0.123 (0.082)	−0.200 * (0.109)	0.043 (0.147)
Northeast	0.366 *** (0.105)	0.001 (0.101)	0.197 * (0.107)	0.227 ** (0.129)	0.361 *** (0.124)	0.896 *** (0.258)
North	0.803 *** (0.102)	0.475 ** (0.193)	0.710 *** (0.161)	0.834 *** (0.092)	0.734 *** (0.120)	0.980 *** (0.197)
Northwest	0.731 *** (0.091)	0.068 (0.121)	0.436 *** (0.132)	0.835 *** (0.097)	0.934 *** (0.110)	1.031 *** (0.175)
South	0.309 *** (0.110)	0.090 (0.133)	0.207 (0.115)	0.281 ** (0.138)	0.304 ** (0.154)	0.368 (0.247)
Southwest	0.462 *** (0.097)	0.047 (0.106)	0.297 ** (0.114)	0.477 *** (0.121)	0.482 *** (0.136)	0.847 *** (0.199)
East	−0.071 *** (0.100)	0.007 (0.090)	0.017 (0.095)	−0.016 (0.102)	−0.114 (0.125)	−0.129 (0.212)
Constant	7.262 *** (0.288)	3.863 *** (0.584)	5.564 *** (0.430)	7.538 *** (0.301)	9.103 *** (0.400)	10.403 *** (0.553)
N	13,784					

Asymptotic standard errors are in parentheses. *** *p* < 0.01, ** *p* < 0.05, * *p* < 0.10.

**Table A3:** Estimated parameter coefficients from the instrumental variable quantile regression model with EP2 as energy poverty measure.

	Quantile Regression				
Variable	q10	q25	Median	q75	q90
Ep2	−0.115 *** (0.029)	−0.060 (0.073)	0.194 *** (0.052)	0.419 *** (0.118)	0.900 *** (0.041)
Age	0.203 *** (0.010)	0.185 *** (0.009)	0.190 *** (0.009)	0.236 *** (0.010)	0.330 *** (0.009)
Household size	0.007 * (0.004)	−0.022 *** (0.005)	−0.036 *** (0.007)	−0.053 *** (0.006)	−0.054 *** (0.004)
Household income	0.000 *** (0.000)	0.000 (0.000)	0.000 *** (0.000)	0.000 (0.000)	0.000 *** (0.000)
Male	−0.502 *** (0.026)	−0.659 *** (0.018)	−0.752 *** (0.023)	−0.724 *** (0.031)	−1.019 *** (0.025)
Primary School	−0.331 *** (0.052)	−0.186 *** (0.068)	−0.043 (0.069)	0.580 *** (0.095)	1.412 *** (0.080)
Middle to high school	−0.191 *** (0.053	−0.083 (0.067)	−0.165 ** (0.064)	0.163 (0.101)	0.473 *** (0.078)
Some college	−0.276 *** (0.059)	−0.146 * (0.076)	−0.238 ** (0.095)	−0.050 (0.112)	−0.049 (0.102)
Religious belief	0.256 *** (0.018)	0.296 *** (0.016)	0.418 *** (0.033)	0.532 *** (0.028)	0.462 *** (0.028)
Single	−0.064 (0.056)	−0.304 *** (0.058)	−0.723 *** (0.081)	−0.997 *** (0.092)	−1.026 *** (0.071)
Married	−0.429 *** (0.029)	−0.515 *** (0.043)	−1.009 *** (0.055)	−1.329 *** (0.070)	−1.705 *** (0.048)
Divorced	−0.598 *** (0.095)	0.129 (0.144)	0.012 (0.124)	−0.235 ** (0.113)	−0.321 *** (0.106)
Employed	−0.110 *** (0.019)	−0.183 *** (0.027)	−0.128 *** (0.037)	−0.187 *** (0.029)	0.038 (0.033)
Northeast	−0.007 (0.021)	0.182 *** (0.040)	0.196 *** (0.054)	0.345 *** (0.045)	0.854 *** (0.046)
North	0.504 *** (0.032)	0.709 *** (0.040)	0.832 *** (0.043)	0.728 *** (0.040)	0.977 *** (0.040)
Northwest	0.060 ** (0.024)	0.430 *** (0.029)	0.833 *** (0.039)	0.911 *** (0.044)	1.032 *** (0.031)
South	0.101 *** (0.028)	0.195 *** (0.040)	0.280 *** (0.056)	0.288 *** (0.053)	0.373 *** (0.045)
Southwest	0.055 ** (0.021)	0.270 *** (0.033)	0.489 *** (0.044)	0.490 *** (0.050)	0.793 *** (0.041)
East	0.000 (0.023)	0.016 (0.040)	−0.020 (0.039)	−0.140 ** (0.055)	−0.125 ** (0.050)
Constant	3.844 *** (0.084)	5.512 *** (0.108)	7.445 *** (0.112)	9.039 *** (0.154)	10.341 *** (0.112)
N	13,784				

Asymptotic standard errors are in parentheses. *** *p* < 0.01, ** *p* < 0.05, * *p* < 0.10.
